# Identification of antinutritional, antioxidant, and antimicrobial activity of plants that cause livestock poisoning in Bojonegoro Regency, Indonesia

**DOI:** 10.14202/vetworld.2022.2131-2140

**Published:** 2022-09-06

**Authors:** Maria Rosaria Odilia, Dhiya Tajhanun Zahra Astika Putri, Antasiswa Windraningtyas Rosetyadewi, Agustina Dwi Wijayanti, Agung Budiyanto, Arvendi Rachma Jadi, Anggi Muhtar Pratama

**Affiliations:** 1 Department of Pharmacology, Faculty of Veterinary Medicine, Universitas Gadjah Mada, Jl. Fauna No. 2 Karangmalang, Yogyakarta, 55281, Indonesia; 2 Department of Reproduction and Obstetrics, Faculty of Veterinary Medicine, Universitas Gadjah Mada, Jl. Fauna No. 2 Karangmalang, Yogyakarta, 55281, Indonesia; 3 Department of Anatomy, Faculty of Veterinary Medicine, Universitas Gadjah Mada, Jl. Fauna No. 2 Karangmalang, Yogyakarta, 55281, Indonesia

**Keywords:** antimicrobial, antinutritional, antioxidant, Bojonegoro regency, livestock, plant poisoning

## Abstract

**Background and Aim:**

The utilization of cassava leaves and peels, ceara rubber leaves, sweet potato leaves, Chinese *Albizia* leaves, and lophatheri leaves from Bojonegoro Regency has led to the poisoning of livestock due to antinutritional factors. Nevertheless, the plants are known to have bioactive components and potential antioxidant and antibacterial activity if appropriately processed. This study aimed to determine the antinutritional compounds as well as the antioxidant and antibacterial potential of these plants responsible for livestock poisoning in the Bojonegoro Regency.

**Materials and Methods:**

Extraction was performed by the maceration method using 70% (v/v) ethanol solvent. The samples were analyzed qualitatively to determine the presence of tannins, alkaloids, oxalates, cardiac glycosides, and cyanogenic glycosides. The antioxidant activity was determined using the 1,1-diphenyl-2-picrylhydrazyl method, while the antimicrobial activity was assessed by different testing concentrations (125, 250, and 500 mg/mL) against *Staphylococcus epidermidis*, *Staphylococcus aureus*, and *Escherichia coli*.

**Results:**

The ethanolic extract of the plants was found to contain antinutritional tannins, alkaloids, cardiac glycosides, and cyanogenic glycosides suspected of causing livestock poisoning. Despite the presence of these antinutrients, all extracts also had antioxidant and antibacterial potential. Cassava peels and sweet potato leaves had the highest antioxidant activity, whereas Chinese *Albizia* leaves had the most potent antibacterial activity.

**Conclusion:**

Cassava leaves and peels, ceara rubber leaves, sweet potato leaves, Chinese *Albizia* leaves, and lophatheri leaves obtained from Bojonegoro Regency and used as agricultural waste contain antinutritional factors but also possess potentially effective antioxidant and antimicrobial components.

## Introduction

Bojonegoro Regency is one of the most significant agricultural and livestock breeding areas in East Java, Indonesia, with a livestock population of 4,520,797 [1]. Hence, most of the local income depends on selling livestock products [2, 3]. Local farmers use agricultural waste, including cassava (*Manihot esculenta* Crantz) leaves and peels, ceara rubber (*Manihot glaziovii* Müll. Arg.) leaves, sweet potato (*Ipomoea batatas* (L.) Lam.) leaves, and Chinese *Albizia* (*Albizia chinensis* [Osbeck] Merr.) leaves to fulfill livestock nutrition needs. These plants are obtainable, especially during the dry season, as they are highly adaptable to the environment. Lophatheri (*Lophatherum gracile* Brongn) is a poisonous plant that is often carried away when grazing and mixed during foraging. Repeated use of agricultural waste and lophatheri leads to poisoning cases in livestock. Most poisoning cases were caused by *L. gracile* Brongn (3.33%) and *M. esculenta* Crantz (2.67%), followed by *A. chinensis* (Osbeck) Merr. (0.22%), and *I. batatas* (L.) Lam. (0.11%). While not all livestock is poisoned, such frequent incidents cause losses to local farmers [4]. The livestock industry of Australia has estimated losses of 50 million Australian dollars due to production shortfalls, stock deaths, and additional management required due to plant poisoning, while in western North America, poisonous plants are reportedly responsible for losses totaling an estimated 500 million United States dollars by the livestock industry [5, 6].

Livestock-provided agricultural waste showed the main poisoning symptoms of bloat, foaming at the mouth, and death. Due to uncertainty about the origin of the poisoning in their livestock, farmers cannot prevent or overcome the problem. Pratama *et al*. [4] suspected that antinutritional plant factors were responsible for the incidence of livestock poisoning in Bojonegoro Regency. This assumption was reinforced by the discovery of antinutritional content such as tannins, alkaloids, oxalates, cardiac glycosides, and cyanogenic glycosides in cassava, cearra rubber, sweet potato, Chinese *Albizia*, and lophatheri [7–16]. However, research on the antinutritional content that can cause poisoning of various livestock in Bojonegoro Regency is lacking.

Through proper processing, agricultural waste also has the potential to be developed as antibacterial and antioxidant alternatives [17, 18]. The bioactive components of cassava, ceara rubber, sweet potato, Chinese *Albizia*, and lophatheri can inhibit free radical activities such as flavonoid and saponin, which can suppress 1,1-diphenyl-2-picrylhydrazyl (DPPH) radicals, hydroxyl radicals, superoxide anion radicals, and nitric oxide and suppress bacterial growth [19–21]. This antibacterial and antioxidant potential has been identified in various studies. Cassava has high antioxidant activity and can inhibit *Staphylococcus epidermidis* and *Propionibacterium acnes* [22, 23]. Ceara rubber has high antioxidant activity [24], and sweet potato has high antioxidant activity and can inhibit the growth of *Streptococcus* spp. and *Staphylococcus aureus* [8, 25, 26]. Chinese *Albizia* has high antioxidant activity and can inhibit *S. aureus*, *Pseudomonas aeruginosa*, *Klebsiella pneumoniae*, and *Escherichia coli* [27, 28]. Lophatheri is known to have antioxidant activity and can inhibit *Streptococcus* spp. [29, 30].

No research has been found regarding the antinutritional content, antioxidant, and antibacterial abilities contained in agricultural waste in Bojonegoro Regency. Therefore, this study aimed to investigate the antinutritional substances that have caused poisoning in livestock while examining the antioxidant and antibacterial activities of agricultural waste used as forage for livestock in Bojonegoro Regency. This research is expected to be the first to provide information on herbal medicine and the development of potential herbal products for animals in Indonesia, especially in Bojonegoro Regency. This study offers preliminary information and a screening test for antinutritional compounds, antioxidant, and antibacterial activity of some plants used as alternative forage in the Bojonegoro Regency. Although quantifying the identified metabolites was not performed, identifying the substantial qualitative variations of antinutritional compounds and the medical potential of these plants will provide a better understanding and consideration for future studies.

## Materials and Methods

### Ethical approval

There are no specific regulations governing research for identification of antinutritional, antioxidant, and antimicrobial activity of plants in Indonesia, therefore This research does not require ethical approval.

### Study period, location, and sample collection

The experiment was conducted from August to October 2021 in Universitas Gadjah Mada, Yogyakarta, Indonesia. Samples of dried leaves and peels of cassava, dried leaves of ceara rubber, sweet potato, chinese *Albizia*, and lophatheri were collected from Bojonegoro Regency, East Java, Indonesia (S6°59–7°37’, E112°25’–112°09’). This area consists of 2,307.79 km^2^ and is located over 110 km from the provincial capital of East Java [3, 4, 31].

### Ethanolic extract preparation

The leaves identified refers to research that has been carried out by Pratama *et al*. [4]. The plants were identified by Anggi Muhtar Pratama, Okti Herawati (Faculty of Veterinary Medicine), and Maryanto (tribal elder) with the help of Aries Bagus Sasongko (Faculty of Biology, Universitas Gadjah Mada). We used ethanol as the solvent for our plant extraction based on a previous study conducted by Golestannejad *et al*. [32] that showed the highest inhibition effects from ethanolic extract of olives (*Olea europaea*) rather than hydroalcoholic extracts on *Streptococcus mutans* growth, acid production, and adhesion. Another study conducted by Gupta *et al*. [33] also showed that *Martynia annua* Linn. ethanolic extracts had greater DPPH radical scavenging activity (RSA) than hydroalcoholic and water extracts. These results could be due to ethanol solvent’s ability to attract more total phenolic and flavonoid content, which has also been evaluated *in vivo* and found to have better hepatoprotective activity than hydroalcoholic extracts from the same plant [34].

The leaves identified refers to research that has been carried out by Pratama *et al*. [4] from Bojonegoro Regency were chopped and blended into powder for the extraction process. About 300 g of fully powdered plants were macerated using 1.500 mL of 70% v/v ethanol for 72 h at room temperature (21.5°C) and stirred continuously. The mixture was then filtered and concentrated for further analysis using a water bath set at 70°C.

### Antinutritional screening

The screening was conducted to find antinutritional components such as tannin, alkaloid, oxalate, cardiac glycoside, and cyanogenic glycoside of the cassava leaves and peels, ceara rubber, sweet potato, Chinese *Albizia*, and lophatheri leaves ethanolic extracts using the following standard methods.

To observe the presence of tannin, 0.3 g of plant extract was dissolved in 10 mL of ethanol. Subsequently, two drops of ferric chloride (FeCl_3_) were added to the solution. Changes in the solution to dark blue or dark green indicate tannin content [35].

Alkaloids were assessed by dissolving 0.01 g of plant extract in 5 mL of HCL. The extracts were then homogenized and filtered. As many as three drops of Mayer’s reagent were added to the extract, and a white precipitate or red color confirmed the presence of alkaloids [36].

An oxalate test was conducted by dissolving 0.3 g of plant extract into 3 mL distilled water and adding 5 drops of glacial acetic acid. A change in color to dark green indicated the presence of oxalate [36].

Identification of cardiac glycoside was performed using the Keller-Kiliani method by dissolving 0.5 g of plant extract in 5 mL of distilled water, followed by adding 2 mL of Keller-Kiliani reagent. A total of 1 mL of sulfuric acid (H_2_SO_4_) was added to the solution. The formation of a brown ring indicated the presence of cardiac glycoside [37].

The cyanogenic glycoside content was analyzed using a picrate paper test by dissolving 0.5 g of plant extract into 1 mL HCL. About 1 mL of chloroform was added to the solution, and the sodium picrate paper was tied to the tube cap. The change of the paper to pink indicated the presence of cyanogenic glycoside [38].

### Radical scavenging activity

In the present study, the radical scavenging or antioxidant activities were evaluated using the DPPH method described by Djeussi *et al*. [39] with several modifications. A 1 mL sample in various concentrations (100 ppm, 200 ppm, 300 ppm, 400 ppm, and 500 ppm) was put into a test tube to which 2 mL of DPPH was added. The sample was then incubated in a dark room for 30 min and dissolved in methanol. To calculate the absorbance of DPPH, a solution was made by mixing 1 mL of DPPH with 4 mL of methanol. The absorbance of DPPH and extract was measured using a spectrophotometer at 517 nm. The percentage of inhibition was then calculated using the following equation:


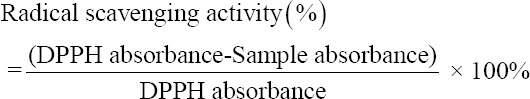


The RSA of plant extracts to inhibit 50% of DPPH free radicals (RSA half maximal inhibitory concentration [IC_50_]) was then calculated based on the linear equation of the results with the percentage RSA value as the vertical axis (Y) and concentration as the horizontal axis (X).

### Determination of antibacterial activity

Antibacterial activity was analyzed using the agar disk diffusion method described by Balouiri *et al*. [40] with several modifications. Each extract was dissolved using dimethyl sulfoxide 10% to obtain concentrations of 125 mg/mL, 250 mg/mL, and 500 mg/mL. Sterile paper disks (6 mm) were then immersed at each concentration for 10–15 min. Inoculum of *S. epidermidis*, *S. aureus* (23SrRNA, isolate code R4H) [41], and *E. coli* was streaked onto Mueller-Hinton agar plates. The disc that had been soaked in the extract was placed on the agar surface and incubated for 24 h at 37°C. Each extract was tested in duplicate with streptomycin (10 μg/mL) as a positive control and a disc with distilled water as a negative control.

### Statistical analysis

The results were analyzed qualitatively using standards from various literatures. The presence of antinutrients was determined by color changes according to María *et al*. [35], Kgosana [36], Ismail *et al*. [37], and Appenteng *et al*. [38]. The RSA was categorized using the IC_50_ range by Djeussi *et al*. [39], with activity considered high if IC_50_ was <50 ppm, moderate at 50–100 ppm, and low if >100 ppm. The antibacterial activity was interpreted based on the size of the inhibition zone of each concentration by classifying the results as weak (<12 mm), moderate (12–20 mm), or high (>2 mm) [42].

## Results and Discussion

### Antinutritional compound

**Figure-1:**
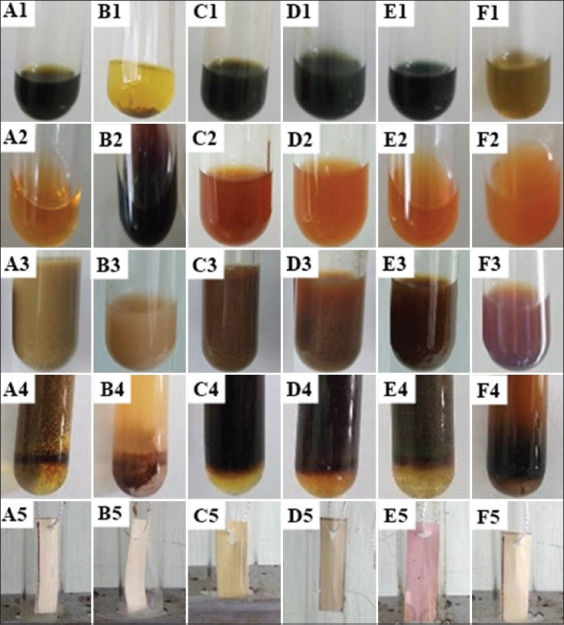
Identifications of antinutritional compounds in various tests. (A) Cassava leaves; (B) cassava peels; (C) ceara rubber leaves; (D) sweet potato leaves; (E) Chinese *Albizia* leaves; (F) lophatheri leaves. For the presence of (1) tannin; (2) alkaloid; (3) oxalate; (4) cardiac glycoside; (5) cyanogenic glycoside.

Antinutritional factors were identified by color changes in the solution and test paper (Figure-1). The plant extract showed the presence of antinutritional alkaloids and cardiac glycosides but no oxalate. Tannin was present in the ethanol extract of cassava leaves, ceara rubber leaves, sweet potato leaves, and Chinese *Albizia* leaves, but not in cassava peels and lophatheri leaves (Table-1).

**Table-1:** Antinutritional components of some plants that cause poisoning in Bojonegoro Regency.

Extracts	Tannin	Alkaloid	Oxalate	Cardiac glycoside	Cyanogenic glycoside
Cassava leaves	+	+	-	+	-
Cassava peels	-	+	-	+	-
Ceara rubber leaves	+	+	-	+	-
Sweet potato leaves	+	+	-	+	-
Chinese *Albizia* leaves	+	+	-	+	+
Lophatheri leaves	-	+	-	+	-

+=Antinutrients were present, -=No presence of antinutrients

A previous study by Okoro [12] found that cassava leaves extracted with various solvents contained alkaloids, tannins, and cardiac glycosides, which is in accordance with this study. Amaza [14] also reported that cassava peel ethanolic extract contained alkaloid, tannin, and cardiac glycoside, as found in the present study, without the presence of tannins. The antinutritional content found in sweet potato leaves extract was in accordance with Osuntokun *et al*. [43]. They also reported that sweet potato leaves ethanolic extract contained alkaloid, tannin, and cardiac glycoside. There have not been many studies related to the antinutritional content in the leave of ceara rubber, lophatheri, and Chinese *Albizia*, but Chikezie *et al*. [9] found the presence of alkaloid and tannin in the methanol extract of cassava leaves, which was also found in this study. Furthermore, alkaloids and cyanogenic glycosides in the ethanol extract of Chinese *Albizia* leaves were also found by Amudha *et al*. [10].

Ismaila *et al*. [11] and Joseph *et al*. [15] stated that the main antinutritional content in cassava plants is cyanogenic glycosides. Interestingly, in this study, the ethanol extract of cassava leaves and bark did not contain any antinutritional cyanogenic glycosides. The undetectability of this content can be due to a decrease in plant antinutritional levels resulting from the extract manufacturing processes such as drying and chopping [44].

Tannins, alkaloids, oxalates, cardiac glycosides, and cyanogenic glycosides are antinutritional factors responsible for various livestock poisoning cases in Bojonegoro Regency. Similar symptoms of poisoning in livestock after consuming agricultural waste were reported by Pratama *et al*. [4] due to the same antinutrients [45, 46]. These findings are similar to Rubini *et al*. [47] and Ceci *et al*. [48], who found various poisoning cases of livestock in Italy due to the cardiac glycoside content in *Nerium oleander*. Melo *et al*. [49] and Prasad *et al*. [50] also reported several livestock poisoning cases due to alkaloids and cyanogenic glycoside-containing plants in Brazil and India. Hypersalivation, convulsions, abortion, bloat, infertility, and even death can be caused by alkaloids [51, 52]. Frothing mouth, convulsions, exophthalmos, and bloat can be caused by cyanogenic glycosides [44, 53, 54]. Moreover, anorexia and lethargy can be caused by tannins and cardiac glycosides [4, 55]. Compounds containing condensed tannins and hydrolyzable tannins are believed to be toxic to livestock, including ruminants [56]. Cassava leaves have been found to contain condensed tannins (cyanidin and delphinidin), which play a harmful role in decreasing protein digestibility [57]. However, various plants could have different condensed tannin protein-binding abilities, which could affect their toxicity. Furthermore, it has been found that the toxicity effect of condensed tannins is inconsistent in plants due to the different structures of the compounds [56]. Hence, the toxicity of tannins in plants could vary and needs further research.

Various post-harvest processes can reduce antinutritional factors in plants. Adegbehingbe *et al*. [58] found that the content of tannin, oxalate, and cyanide in *Albizia lebbeck* seeds can be significantly reduced by fermentation. A similar study found that the fermentation of sweet potato leaf meal by *Chaetomium globosum* could reduce antinutritional factors such as tannin, phytates, trypsin inhibitors, alkaloids, and oxalates [59]. Agbai *et al*. [60] added that fermentation and boiling processes could reduce tannins, alkaloids, oxalates, and hydrogen cyanide in *Hevea brasiliensis* seeds. Chopping, drying, soaking, germination, and cooking were also found to reduce antinutritional factors such as tannins, alkaloids, cyanides, phytates, and oxalates in various plants [61–63]. Furthermore, combining two or more processing methods can significantly reduce plant antinutritionals [61].

### Antioxidant activity

Extracts from cassava leaves and peels, ceara rubber leaves, sweet potato leaves, Chinese *Albizia* leaves, and lophatheri leaves were potentially effective in reducing radical activity with various strengths (Table-2). Among the six extracts tested, only cassava peels and sweet potato leaves showed high antioxidant activity, followed by cassava leaves and Chinese *Albizia* leaves with medium activity, and ceara rubber leaves and lophatheri leaves with low activity.

**Table-2:** Antioxidant activity of the ethanolic extract of some plants that cause poisoning in Bojonegoro Regency.

Plant extracts	Antioxidant activity
Cassava leaves	++
Cassava peels	+++
Ceara rubber leaves	+
Sweet potato leaves	+++
Chinese *Albizia* leaves	++
Lophatheri leaves	+

+++=High activity (IC50<_50_ ppm), ++=Medium activity (50 ppm<IC_50_<100 ppm), +=Low activity (IC_50_>100 ppm), IC_50_=Half maximal inhibitory concentration

Based on the present study results, the lowest DPPH IC_50_ of the plant extracts showed high scavenging activity. This is in accordance with Lachkar *et al*. [64], who reported a lower IC_50_ level of an extract corresponds to higher antioxidant activity. The high antioxidant activity of extracts of cassava peels and sweet potato leaves is in accordance with the results of research conducted by Lateef *et al*. [22] and Zhang *et al*. [65], who found that the two plant extracts showed high antiradical activity. However, the antioxidant abilities of cassava leaves, ceara rubber leaves, Chinese *Albizia* leaves, and lophatheri leaves, while still lower than the previous studies, were found to have high antioxidant activity [12, 24, 27, 66]. Differences between the present study results and those reported in the literature can be due to variations in the concentration and type of solvent, extraction method, amount of total polyphenol and total flavonoid content, environmental factors, and treatment during plant growth [67–70].

The ability of plants to inhibit free radical activity is due to various phytochemical substances, mainly total polyphenol content and total flavonoid content [69]. Polyphenols have mechanisms to provide hydrogen donors, heavy metal chelators, and electrons or increase the response of antiradical cells to reactive oxygen species [71–73]. Alam *et al*. [72] found cassava to have the highest total polyphenol content among other Bangladeshi vegetables. This could be attributed to the high scavenging activity of cassava extracts among other crops tested. Similar results were also reported in sweet potato leaves [74]. However, variations in flavonoid and polyphenol components can be influenced by genetic and biological variations; therefore, the results of antioxidant activity in each plant can be different [75].

Despite coming from the same type of plant, antioxidants in cassava leaves and peel had different levels. Similar results have also been reported by Ekeledo *et al*. [76], who found that yellow cassava peel extract had higher scavenging activity than cassava stem extract. Gonçalves *et al*. [77] also found differences in antioxidant activity in the leaves, seeds, pulp, and peel of *Chamaerops humilis* L. These differences could be caused by alterations in the structure of the phenolic components in plants due to variations in cell structure and chemical substances in plant tissues.

### Antibacterial activity

**Figure-2:**
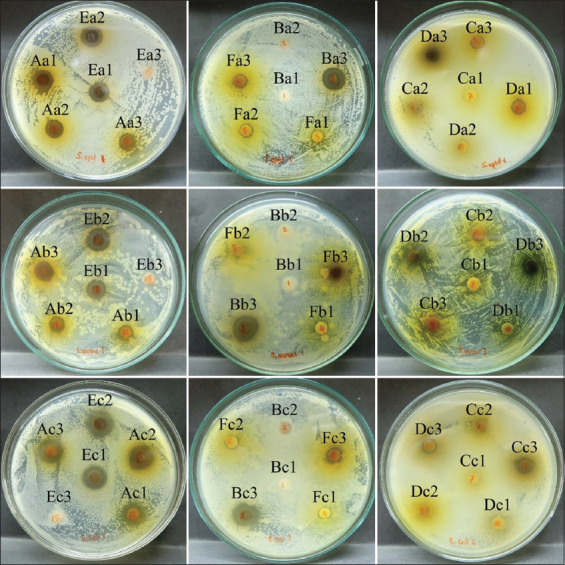
Identification of antibacterial activity in the agar plates. (A) Cassava leaves; (B) cassava peels; (C) ceara rubber leaves; (D) sweet potato leaves; (E) Chinese *Albizia* leaves; (F) lophatheri leaves; (1) 125 mg/mL; (2) 250 mg/mL; (3) 500 mg/mL against (a) *Staphylococcus epidermidis;* (b) *Staphylococcus aureus*; (c) *Escherichia coli*.

Each plant extract showed a different inhibition zone diameter (Figure-2); therefore, it can be concluded that each plant has different antibacterial activity. Based on the interpretation of the inhibition zone, the six samples tested had a broad-spectrum antibacterial activity of weak to medium strength (Table-3). The best ability to inhibit *S. epidermidis* growth was shown by extracts of cassava leaves (500 mg/mL), cassava peel (500 mg/mL), and Chinese *Albizia* leaves (250 and 500 mg/mL). Extracts of cassava peel (500 mg/mL) and Chinese *Albizia* leaves (250 and 500 mg/mL) also showed the most potent inhibitory activity against *S. aureus* and were equal to the standard antibiotic used. Meanwhile, the most significant inhibition of *E. coli* equal to the antibiotic control was from extracts of cassava leaves (125, 250, and 500 mg/mL), cassava peel (500 mg/mL), and Chinese *Albizia* leaves (125, 250, and 500 mg/mL). However, most plant extracts showed lower sensitivity than streptomycin (10 μg/disc).

**Table-3:** Antibacterial activity of the ethanolic extract of some plants that cause poisoning in Bojonegoro Regency.

Plant extracts	Concentration (mg/mL)	Antibacterial activity		

		*Staphylococcus epidermidis*	*Staphylococcus aureus*	*Escherichia coli*
Cassava leaves	125	+	+	++
	250	+	+	++
	500	++	+	++
Cassava peels	125	-	-	-
	250	-	-	-
	500	++	++	++
Ceara rubber leaves	125	+	+	+
	250	+	+	+
	500	+	+	+
Sweet potato leaves	125	+	+	+
	250	+	+	+
	500	+	+	+
Chinese *Albizia* leaves	125	+	+	++
	250	++	++	++
	500	++	++	++
Lophatheri leaves	125	+	+	+
	250	+	+	+
	500	+	+	+
Streptomycin	10mg	+++	++	++
Empty disc		-	-	-

-=Inactive (6 mm), +=Low activity (<12 mm), ++=Intermediate activity (12–20 mm), +++=Strong activity (>20 mm)

The inhibition of bacterial growth from plant extracts results from various bioactive components such as tannins, alkaloids, flavonoids, saponins, and monoterpenes that can disrupt cell structure and interfere with enzyme synthesis and cell permeability, inhibit efflux pumps, and affect energy formation [78]. Interestingly, the presence of these components is thought to cause the plants to have antibacterial activity. As shown in the antinutrients tested (Table-1), some plants contain alkaloids and tannins. In addition, other studies also proved the presence of flavonoids, saponins, and monoterpenes in the six plant extracts tested [23, 78–81].

Based on the bacterial inhibition zone, a higher concentration of an extract provided better antibacterial activity. Therefore, the bacterial inhibition zone indicated increased activity of cassava leaves against *S. epidermidis* and Chinese *Albizia* leaves against *S. epidermidis* and *S. aureus*. El Feghali *et al*. [82] also found a widening of the inhibition zone after the concentration of *Curcuma longa*, *Opuntia ficus-indica*, and *Linum usitatissimum* extracts were increased. With increasing concentrations of plant extracts, more bioactive components can be extracted, resulting in more bacterial cell damage [83–85], which causes bacteria to be unable to grow and widens the size of the inhibition zone [23].

Furthermore, the present study showed that the extracts of cassava leaves, cassava peels, and Chinese *Albizia* leaves had antibacterial activity equivalent to the streptomycin positive control against *E. coli* and *S. aureus*. As such, these three plants have the best potential as antimicrobials. This finding is similar to that of Mehta *et al*. [86], who also found that eucalyptus essential oil and *Mentha* spp. extracts have better antimicrobial activity than standard antibiotics. Another study by Kebede *et al*. [87] found that various herbal plant extracts have the potential as antibiotics to deal with multi-drug-resistant bacteria such as *S. aureus*, *Streptococcus pyogenes*, *E. coli*, and *K. pneumoniae*. This potential can be caused by bioactive components in plants that can disrupt the membranes in bacteria, making them sensitive to extracts [88]. In addition, bioactive components can also inhibit enzyme synthesis, efflux pumps, and energy formation, which weakens the bacterium’s activity [78]. This ability can be increased by increasing the concentration of the extract, which can also affect the presence of bioactive components; therefore, plant extracts could have better antibacterial sensitivity at a particular concentration than standard antibiotics [86, 89].

## Conclusion

This research indicates that the extracts of cassava leaves, cassava peels, ceara rubber leaves, sweet potato leaves, Chinese *Albizia* leaves, and lophatheri leaves obtained from Bojonegoro Regency, used as agricultural waste, contained antinutritional factors but also possess potentially effective antioxidant and antimicrobial components. The observed data bring new important information regarding plant extracts’ antioxidant and antimicrobial potential, which can be utilized as herbal medicines with proper processing. However, there is a lack of knowledge regarding plant extracts’ toxicity and protective effect on living organisms; therefore, further studies are required to evaluate the significant impact of plant actions *in vivo* before their development and use as herbal medicines.

## Authors’ Contributions

All authors contributed to the study design, interpreted the data, and wrote the manuscript. MRO and DTZAI: Processed the samples, performed antinutritional, antibacterial, and antioxidant tests. AMP, AWR, ADW, AB, and ARJ: Reviewed and edited the draft. All authors have read and approved the final manuscript.
